# Ultrasound to address medullary sponge kidney: a retrospective study

**DOI:** 10.1186/s12882-020-02084-1

**Published:** 2020-10-12

**Authors:** Isabella Pisani, Roberto Giacosa, Sara Giuliotti, Dario Moretto, Giuseppe Regolisti, Chiara Cantarelli, Augusto Vaglio, Enrico Fiaccadori, Lucio Manenti

**Affiliations:** 1 U.O. Nefrologia, Azienda Ospedaliero-Universitaria di Parma, Dipartimento di Medicina e Chirurgia, Università di Parma, Via Gramsci 14, 43126 Parma, Italy; 2 Casa di Cura Città di Parma, Unit of Diagnostic, Contrast enhanced and Interventional Ultrasound, Parma, Italy; 3 Struttura complessa di Radiologia, Azienda Ospedaliero-universitaria di Parma, Parma, Italy; 4 Department of Biomedical Experimental and Clinical Sciences “Mario Serio”, University of Firenze, Florence, Italy; 5 Nephrology and Dialysis Unit, Meyer Children’s University Hospital, Florence, Italy

**Keywords:** Renal cystic disease, Ultrasonography, Medullary sponge kidney, Nephrolithiasis, Chronic renal failure

## Abstract

**Background:**

Medullary sponge kidney (MSK) is a rare disease characterized by cystic dilatation of papillary collecting ducts. Intravenous urography is still considered the gold standard for diagnosis. We identified a cohort of patients from our outpatient clinic with established diagnosis of MSK to outline some ultrasonographic characteristics that may help establish a diagnosis.

**Methods:**

We conducted a retrospective study of patients seen between January 1st 2009 and January 1st 2019 in our clinic. Out of 4321 patients, 18 had a diagnosis of MSK. We reviewed their clinical and family history, laboratory data and imaging studies. Specifically, we focused on ultrasound imaging.

**Results:**

Patients were referred to our outpatient clinic because of renal impairment (44%), family history of nephropathy (17%), nephrolithiasis or an established diagnosis of MSK (39%). Seventy-two percent of patients presented with chronic kidney disease, 22% required hemodialysis. Urinary tract infections (44%), nephrolithiasis (33%), microscopic hematuria (50%) and proteinuria (44%) were reported. Seven patients underwent computed tomography; all of them received ultrasound. Ultrasound examination showed bilateral renal cysts, usually small and located in the renal medulla, and microcalcifications located in the medulla or within the cysts.

**Conclusion:**

We identified a peculiar tetrad associated with MSK: 1) hypoechoic medullary areas, 2) hyperechoic spots, 3) microcystic dilatation of papillary zone, 4) multiple calcifications (linear, small stones or calcified intracystic sediment) in each papilla. The presence of this diagnostic tetrad, added to laboratory data and clinical history, could be helpful in the differential diagnosis to identify patients with MSK.

## Background

Medullary sponge kidney (MSK) is a rare renal disease, characterized by ectasia and cystic dilatation of intrapapillary portions of medullary collecting ducts that give the renal medulla a “spongy” appearance at autopsy. Its prevalence in the general population is not exactly known, but is estimated to be about 1/5000 persons and, among patients with recurrent nephrolithiasis, it ranges from 12 to 20% [1–7]. MSK is typically associated with nephrocalcinosis and recurrent renal stones formation, distal renal tubular acidosis, hypocitraturia, hypercalcemia, renal concentration defects and defects of the proximal tubule, such as low molecular weight proteinuria and an altered Tm (transport maximum) for glucose, phosphate and para-aminohippuric acid [1, 2, 7].

Intravenous urography (IVU) is still considered the gold standard for the diagnosis of MSK. This technique reveals pathognomonic images of collection of contrast medium in dilated papillary ducts, giving the appearance of a blush or linear striations in the mildest cases or of a bouquet of flowers in full-blown cases [1–4, 6, 8–10]. However, as in patients with nephrolithiasis urography is now less used in favor of computed tomography (CT), the chances to diagnose MSK are likely reduced [11]. In fact, neither ultrasonography, nor CT or magnetic resonance (MR) are thought to provide specific findings for the diagnosis of the disease [1, 2, 8]. Nevertheless, recognizing MSK is clinically important because – even if there is no specific therapy- these patients should receive prophylaxis against stone formation and urinary tract infections to avoid progression to renal failure [1, 2, 5–7].

Herein, we analyzed a cohort of patients with an established diagnosis of MSK to define ultrasonographic characteristics that can help establish a correct diagnosis.

## Methods

After obtaining the approval of the Institutional Review Board, we conducted a retrospective study of patients evaluated at the Outpatient Clinic of the Nephrology Department of the University Hospital of Parma from January 1st, 2009 through January 1st 2019. We screened the “Diagnosis” field of the 4321 patients whose medical records were stored in our computer system and we identified 18 patients highly suspected for MSK. After patients had given informed consent, data were collected from medical reports of outpatient visits, with particular attention to the following points: previous medical history, presence of a family history of kidney disease, presence of comorbidities that can affect renal function, age of referral or diagnosis of MSK, symptoms compatible with MSK, the imaging and laboratory analysis performed.

We reviewed all reports of imaging studies, including CT, ultrasound (US) and x-ray, and the indications to perform the above studies, with particular attention to the description of kidney morphology: the pole-to-pole diameter, the presence of cysts, their location (cortical, medullary or both) and size, the presence of renal stones or parenchymal calcifications, along with their size and location. Finally, the diagnosis suggested by the radiologist was recorded. If more studies of the same type were available, analyzing them, we gave particular attention to the most recent. So, even if patients began their follow up over a period of 10 years, the imaging studies reviewed belonged to a shorter period of 3 years increasing their comparability. All ultrasonographic data were obtained from existing records and were reviewed by an expert sonographer with 20 years of experience (R.G.), whereas CT images were reevaluated by a radiologist with 15 years of experience (G.S.)

Biochemical data were also evaluated, with particular attention to serum creatinine and eGFR (estimated glomerular filtration rate), urinalysis for the presence of proteinuria and microscopic hematuria, and quantitative measurements of 24-h urine excretion of sodium, potassium, calcium, phosphate and citrate to assess the individual risk of kidney stone formation.

Checking patients laboratory and clinical data other causes of nephrocalcinosis are excluded.

### Statistical analysis

Only descriptive statistics were performed, with continuous variables being reported as mean (SD) or median (range) as appropriate, and categorical variables being reported as n (%).

## Results

### Clinical and biochemical data

Fourty-4 % of the patients included in the study was started on nephrological follow-up because of the detection of increased serum creatinine at routine laboratory analysis, 17% was referred because of a family history of kidney disease, 39% because of a previous diagnosis of MSK, nephrocalcinosis or because of recurrent episodes of nephrolithiasis. Overall, there were 5 patients (28%) with normal kidney function, 13 (72%) with chronic kidney disease (CKD) at different stages; among these patients 5 (the 28% of the cohort) reached the end-stage renal disease (ESRD) and of them 2 (11%) were on hemodialysis and 3 (17%) had received a kidney transplant (Tab. 1).

**Table 1:** Clinical and ultrasound characteristics of the patients

Characteristic	Value (*n* = 18)
Men/Women (n/n)	4/14
Age –mean y (range)	50 (28–82)
Family history –no. (%)	10 (56)
Prognosis-no.(%)	
Normal kidney function	5 (28)
CKD	13 (72)
Hemodialysis	2 (11)
Kidney transplant	3 (17)
Clinical signs and symptoms-no. (%)	
Microhematuria	9 (50)
Proteinuria	8 (44)
Urinary tract infection	6 (33)
Renal colic	6 (33)
Asymptomatic	2 (11)
Not available	1 (5.5)
Imaging study-no. (%)	
Intravenous urography	0 (0)
CT	7 (39)
Ultrasound	18 (100)
Renal cysts characteristics at ultrasound	
Unilateral/bilateral (n/n)	2/15
Not described (n)	1
Medullary (n)	6
Cortical (n)	1
Cortical and medullary (n)	5
Parenchymal (n)	3
Not described (n)	3
Diameter of medullary cysts (mm)	From 1 mm to 30 mm
Diameter of cortical cysts (mm)	From 25 mm to 65 mm
Renal calcifications or renal stones-no. (%)	16 (89)
Indication for ultrasound-no. (%)	
Follow up of CKD	8 (44)
Follow up of MSK or renal stones	7 (39)
Family history of renal disease	3 (17)
Relevant comorbidities (n)	
Diabetes	3
Nephrectomy	1
Hypertension	1

*CKD* Chronic kidney disease, *CT* Computed tomography, *MSK* Medullary sponge kidney, *y* Years

Ten patients (56%), belonging to two different families, had a family history of kidney disease, characterized by autosomal dominant inheritance; among them, the incidence of ESRD was highest: in the first family, 3 members began hemodialysis and 2 of them received kidney transplant; in the second one a patient is still on hemodialysis.

The diagnosis of MSK was always established in adulthood (age ranging from 28 to 82 years).

Apart from CKD, 33% of patients presented with recurrent renal stones and the 44% had urinary tract infections (UTI). Proteinuria was documented in 44% of cases, ranging from mild (about 0.5 g/day) to nephrotic range (> 3.5 g/day). Microscopic hematuria was detected in 50% of cases and one patient had episodes of macroscopic hematuria, during renal stone colic. Signs and symptoms could present with different combinations: only two patients had the entire spectrum, such as proteinuria, microhematuria, renal stones and UTI, three displayed a combination of renal stones and UTI and three of proteinuria and microhematuria. Moreover, three patients complained only one sign/symptom (isolated proteinuria or isolated microhematuria or renal stones). Only 2 patients (11%) were asymptomatic: one of them was diagnosed because of family history of MSK and the other during follow-up after unilateral nephrectomy. Of 1 patient data about signs and symptoms were not available. The others referred two signs/symptoms combined in different ways.

The analysis of urinary electrolytes, performed in 4 patients, revealed hypocitraturia and low potassium excretion.

As for comorbidities, 3 patients (17%) presented with diabetes mellitus, one (6%) underwent unilateral nephrectomy because of benign renal tumor, two (11%) had mild hypertension. Among them diabetes was constantly present in patients with also CKD or ESRD, but in only one case it could be responsible of renal impairment (renal biopsy gave a diagnosis of diabetic nephropathy), in the other two patients we had no signs of renal involvement (absence of proteinuria and of microvascular complications like diabetic retinopathy). The 67% had no comorbidities that could affect kidney function (Table. 1). For what concern metabolic bone disease, two patients presented osteoporosis. Clinical data are detailed in Supplementary Table 1.

### Imaging studies

All patients underwent US examination at the beginning of nephrological follow-up, and US imaging was repeated yearly in the majority of them (72%) to check the presence of renal stones and look for changes in cyst appearance. We identified three major reasons for prescribing US: follow-up of CKD (44%), a family history of kidney disease (17%) and follow-up of renal stones or renal calcifications (39%).

Seven patients (39%) underwent CT, 5 without use of intravenous contrast medium for the detection of renal stones during a renal colic and 2 with mean of contrast. Only two (11%) of them were examined with abdomen X-ray to screen for vascular calcifications in the setting of kidney transplant.

For what concern diagnosis in one case the radiologist performing CT reported a diagnosis of MSK; in the other cases CT records describe multiple small calcifications within the renal parenchyma or the presence of renal stones with or without the concomitant description of cysts, but a specific diagnosis was not suggested. When CT studies were revised by the expert radiologist only one showed the typical CT-appearance of MSK, whereas the others, could detect cysts and calcifications or renal stones without addressing a specific diagnosis., probably mainly because of the lack of contrast media or of the specific urographic sequence.

On the other hand, US examination suggested a diagnosis of MSK in 13 patients (72%), nephronophtisis in one (6%) and an overlap between MSK and medullary cystic disease in 4 cases (22%).

### Ultrasound imaging

Kidney size, as evaluated by pole-to-pole diameter, parenchymal echogenicity and cortico-medullary differentiation were variable according to the degree of chronic kidney disease.

**Fig. 1:**
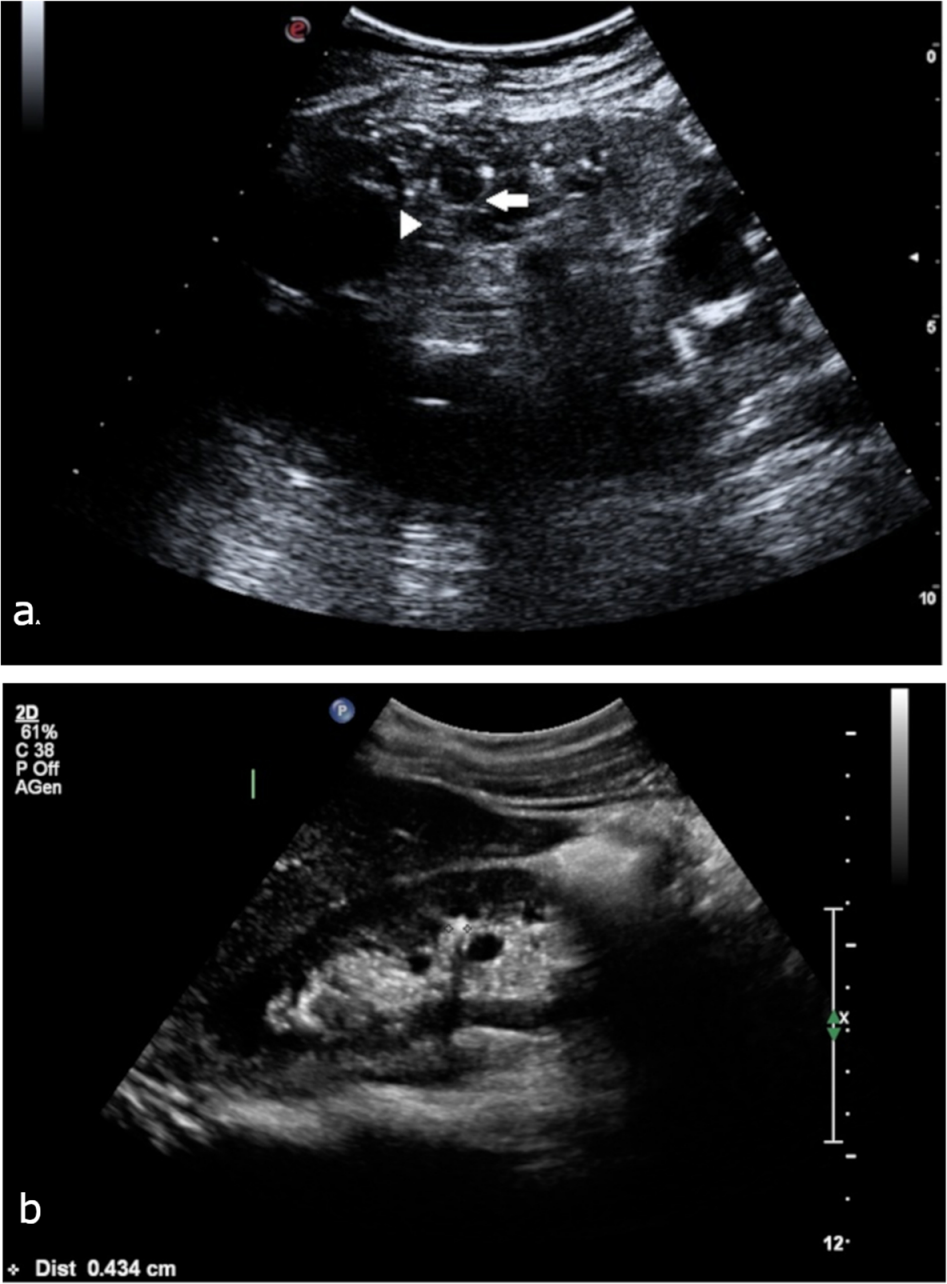
**a** B-mode image representing the right kidney, scanned with a 5.0 MHz convex probe. Note the several cystic dilations of the inner medulla (white arrow) and segmental linear hypercoic strands (arrowhead) (Esaote MyLab Seven, 5.0 MHz convex probe); **b** Renal stone (4 mm) in the mid calyx and parenchimal cyst in a patient with MSK (Philips iU 22, multifrequency convex probe C5–1)

**Fig. 2:**
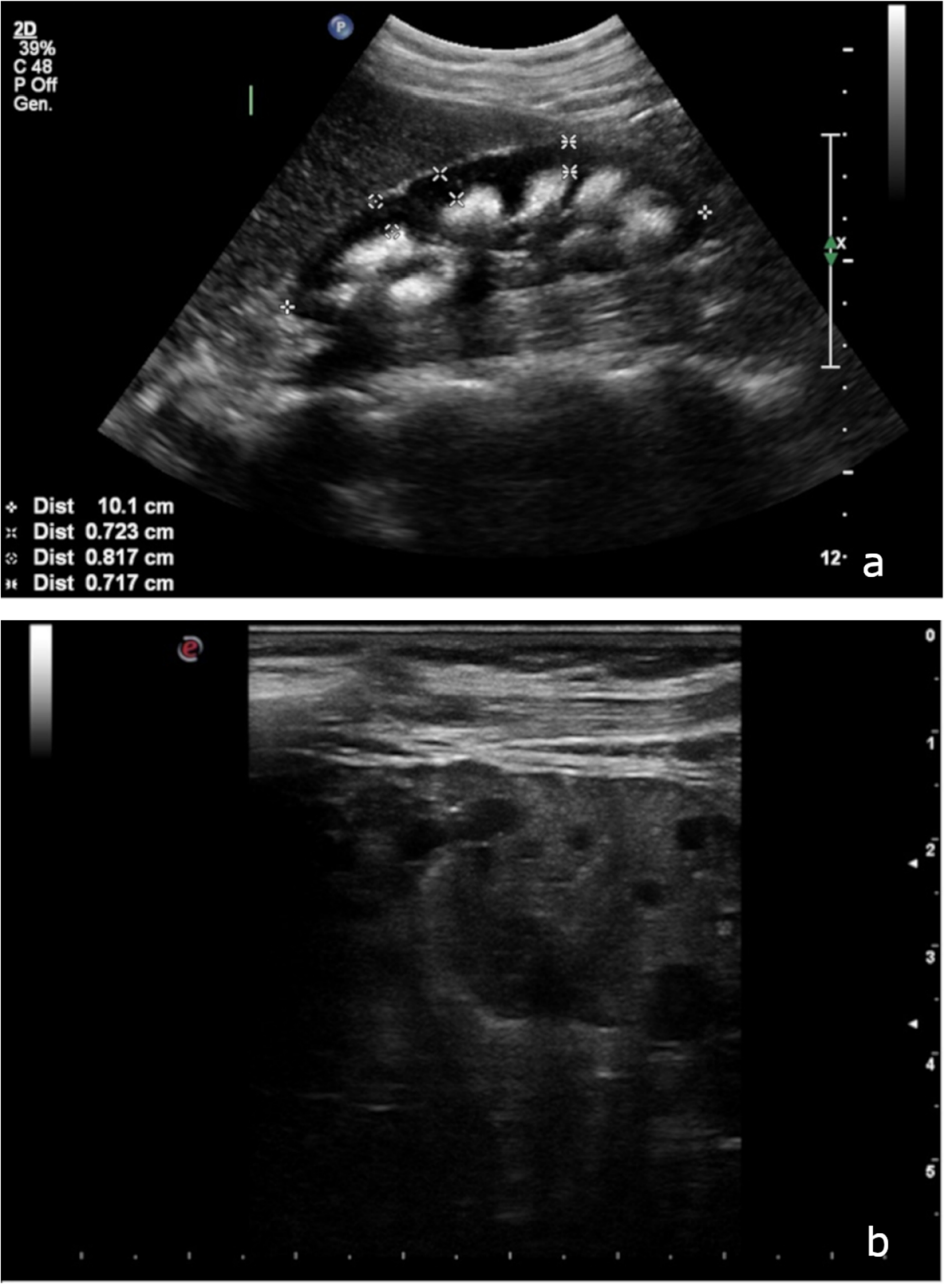
**a** Right kidney showing inversion of the normal cortico-medullary echogenicity pattern: in this patient with nephrocalcinosis, the pyramids and medulla are strikingly hyperechogenic, because of the deposition of crystals (Philips iU 22, multifrequency convex probe C5–1); **b** B-mode image of the right kidney, obtained with a 12.5 MHz linear probe. Cysts of the inner medulla are better recognized (Esaote MyLab Seven, 12.5 MHz linear probe)

Sixteen out of 17 patients (94%) showed bilateral cysts (one patient had previously undergone unilateral nephrectomy). Cysts were usually small (diameter ranging from 1 mm to 30 mm), sometimes referred to as microcysts, and were usually located in the medullary or papillary areas of the kidneys (Fig. 1a). Six patients (33%) also had cortical cysts that were larger than the medullary ones (diameter ranging from 25 to 65 mm). Microcalcifications were also frequently reported (16 patients, 89%), and were frequently described as ‘hyperechoic spots’, ‘hyperechoic lines’ or frank renal stones with a diameter ranging from 3 to 10 mm. The calcifications were usually localized in the renal medulla, in close relationship with the medullary cysts, and were frequently described as papillary/medullary calcifications or calcific deposits within the cysts (Fig. 1b) (Table. 1). In some cases the description reported a peripheral hyperechoic aspect of medulla due to multiple microcalcific deposition named nephrocalcinosis (Fig. 2a). The ultrasound exams were conducted both with a convex 1–5 MHz probe and with a higher frequency linear probe (9–3 and also 12–5 MHz) (Fig. 2b). Data about renal ultrasonography are detailed in Supplemetary Table 2.

## Discussion

MSK was recognized as a distinct disease with the introduction of urography in clinical practice. However, this pathological entity seems to be disappearing, likely because urography has almost been abandoned in favor of abdominal CT, usually without administration of contrast medium, which lacks specific/pathognomonic signs of MSK [1, 2, 4, 6, 12]. In fact unenhanced abdominal CT has replaced IVU in the diagnostic work-up of acute renal colic from ureteral calculi because of a higher sensitivity and specificity [11]. This scenario was confirmed by the analysis of our cohort, in which intravenous urography was not available for review and CT was mainly performed without contrast medium (for a study of kidney stones) limiting its resolution power. The use of contrast medium-enhanced CT urography (CTU) with 3D volume-rendered imaging could be useful to diagnose MSK [10, 13, 14]. There are two studies published about using CTU to diagnose MSK. The first one is a series of 15 patients that underwent CTU because of a recurrent symptomatic nephrolithiasis. Four of them presented the characteristic radiologic findings of MSK (collecting tubules dilatation, medullary nephrocalcinosis, nephrolithiasis and medullary cysts) [15]. The second is a trial that compared IVU and multidetector CTU for diagnosis of MSK in 10 patients. It demonstrated that multidetector CTU had a sensitivity of 90% and a specificity of 100% when compared with IVU [16]. However, the routine use of this imaging technique has some limitations in patients and in particular in renal ones. First of all, there is a need to reduce the use of radiocontrast media in the presence of CKD (like in our cohort), due to the potential risk of contrast-induced nephropathy. Secondly, CT urography could not be ethically proposed as a mean of screening for asymptomatic family members. Finally, it cannot be repeated frequently as a follow-up imaging method, due to a substantial patient exposure to radiations [8]. The previous cited studies, proposing multidetector CTU, lacked considerations about patients renal function as a possible limitation in using contrast media and only one of them reflected about the opportunity to use dose reduction protocol to limit patients radiation exposures [15, 16].

In contrast with CT use, we found that there was an extensive use of US imaging in our patients, in most cases with a dedicated operator. The typical ultrasonographic description of MSK is the following: hypoechoic medullary areas with hyperechoic spots and microcystic dilatation of papillary zone, with multiple calcifications being detected in each papilla. These calcifications can be described in different ways (linear, spots, small stones) or can assume the aspect of nephrocalcinosis. Nephrocalcinosis is usually the only US feature described in literature as being associated with MSK [1, 2, 17, 18]. In our cohort, these alterations were almost always bilateral and considering the usual presence of microcysts and microcalcifications when the presence of MSK is suspected, it would be recommended to complete the US examination with a high-frequency linear probe to increase exam sensitivity. We would like also to point out that the presence of cortical cysts, as displayed by some of our patients, does not exclude the diagnosis of MSK because, once autosomal dominant polycystic kidney disease (ADPKD) and multicystic dysplastic kidney disease have been ruled out, cortical cysts could be simply related to aspecific kidney degenerative changes associated with aging or progression of CKD [19–22]. ADPKD can be recognized because of bilateral enlarged kidneys with multiple cysts of variable size, with cortical and medullary distribution and increasing with time; the multicystic dysplastic kidney disease is always unilateral, detected at birth, with peripherally located cysts with a central region of solid tissue and absence of renal vessels and pelvicalyceal system [18–23]. Moreover, as cysts could be the expression of kidney aging, it is also important to rule out the so called acquired cystic kidney disease, an acquired disorder without any kind of familial clustering, that is characterized by small, hyperechoic kidneys with cysts and is diagnosed in patients with ESRD [20, 21] (Table. 2).

**Table 2:** Main differential diagnosis between medullary sponge kidney and other cystic kidney diseases

Diseases	Bilateral/Unilateral	Kidney size	Ultrasound appearance of kidneys and cysts	Hereditary/acquired	Genetics	Clinical findings
**Autosomal dominant polycystic kidney disease (ADPKD)** [15, 18, 24, 25]	Bilateral	Increased	Presence of multiple, variably sized, cortical and medullary cysts, that increase in number and size with time	Hereditary (autosomal dominant)	PKD1, PKD2, GANAB	Chronic kidney disease typically occurs in adulthood; cysts could be found in other organs (eg: liver, pancreas, seminal vesicles); possible presence of intracranial aneurysms
**Medullary cystic kidney disease (MCKD)/ Autosomal dominant tubulointerstitial kidney disease (ADTKD)** [16, 18, 26]	Bilateral	Small to normal size kidneys	Multiple cysts in the medulla and at the cortico-medullary junction with cortical sparing	Herditary (autosomal dominant)	MCKD1 (now MUC1), MCKD2	Chronic renal failure manifesting in adulthood; no associated syndrome
**Nephronophtisis (NPH)** [15, 16, 21, 24]	Bilateral	Moderately enlarged (infantile NPH); small to normal size (juvenile and adolescent/adult NPH)	Small, hyperechoic kidneys with loss of cortico-medullary differentiation; progressive cystic disease with numerous small discrete cysts in the medulla and cortico-medullary junction	Hereditary (autosomal recessive)	More than ten mutations identified until now (eg; NPHP 1 e NPHP4)	Polyuria and polydipsia because of renal concentration defect; growth retardation; chronic anemia resistant to therapy; chronic renal failure with end-stage renal disease developing in infancy or at a median age of 13 years (juvenile NPH) or 19 (adolescent/adult NPH). Presence of associated syndromes
**Multicystic dysplastic kidney** [15, 17, 18]	Unilateral	Small kidney that disappear in adulthood	Kidney replaced by noncommunicating cysts with a central region of soft tissue; absence or severe atrophy of ipsilateral ureter, renal collecting system and renal vasculature	Acquired	None	Often detected in utero or infancy; possible association with contralateral vescico-ureteral reflux (5–43% of cases)
**Acquired cystic kidney disease** [17, 18]	Bilateral	Small	Atrophic hyperechoic kidneys with cysts (at least three in each kidney) varying in size and complexity	Acquired	None	Presence of end-stage renal disease, the incidence increases with the length of time on dialysis; cysts could be complicated (hemorrhage, nephrolithiasis); possible development of renal malignancy
**Medullary sponge kidney (MSK)** [1, 2, 15, 18, 23]	Generally bilateral, mild cases can be diagnosed as unilateral by ultrasound	Normal (size is more linked to the presence or absence of chronic kidney disease)	Hypoechoic medullary areas with hyperechoic spots and microcystic dilatation of papillary zone; multiple calcifications (linear, small stones or calcified intracystic sediment) in each papilla. In some cases nephrocalcinosis could be described	Hereditary in a half of cases (autosomal dominant)	Mutations of GDNF (12% of cases). Genetics under investigation	Typically observed in renal stone formers; possible presentation as pielonephritis; michroematuria and episods of machroematuria associated with nephrolithiasis. Possible presence of hyperparatiroidism. Association with tubular defect (eg: distal renal tubular acidosis, hypocitraturia defective urinary concentration, an altered Tm (transport maximum) for glucose, phosphate and para-aminohippuric acid, low molecular weight proteinuria).

As we observed in our patients, the general aspect of the kidneys, in terms of diameter, parenchymal echogenicity and corticomedullary differentiation is not related to MSK but to the presence of CKD.

Based on reports that were available for our patients, the US specialist can suggest two different diagnoses, namely nephronophtisis and medullary cystic kidney disease (MCKD, redefined as ADTKD, autosomal dominant tubulointerstitial kidney disease in 2015) [26], instead of MSK. In fact, their ultrasound appearance can be very similar to that of MSK. In nephronophtisis kidneys are small to normal in size, with increased echogenicity, reduced corticomedullary differentiation, and renal cyst formation on the corticomedullary border. In MCKD kidneys are normal to small in size, with multiple cysts at the corticomedullary junction and sometimes in the renal medulla. Nevertheless, renal stones and microcalcifications, as well as the mainly medullary distribution of cyst, are not usually detected in these cases [18–21, 27]. Moreover, the clinical presentation is different, as patients with nephronophtisis typically present with polydipsia and polyuria, growth retardation or chronic iron-resistant anemia and develop ESRD in childhood or adolescence, while patients with MCKD present with polyuria and polydipsia, usually develop hyperuricemia and gout and have no history of nephrolithiasis [19, 20, 27].

Clinical history and laboratory findings in our cohort supported the diagnosis of MSK. Particularly, the diagnosis in adulthood, the presence of recurrent nephrolithiasis or of nephrocalcinosis and the detection of hypercalciuria and hypocitraturia. Moreover, the absence of polyuria and polydipsia, hyperuricemia, diagnosis during childhood or unilateral involvement with contralateral kidney hypertrophy could exclude different nephropathies (nephronophtisis, MCKD, multicystic dysplastic kidney disease). Considering the absence of clinical symptoms that could be highly suggestive of MCKD (such as hyperuricemia and gout (ADTKD-UMOD), anemia in childhood or adolescence (ADTKD-REN), MODY5 (ADTKD-HNF1β) or nephronophtisis (polyuria, polydipsia, anemia during childhood)) we decided not to perform a genetic analysis aimed at diagnose these diseases.

Thus, the presence of this ultrasonographic tetrad: 1) hypoechoic medullary areas, 2) hyperechoic spots, 3) microcystic dilatation of papillary zone, 4) multiple calcifications (linear, small stones or calcified intracystic sediment) in each papilla, together with the analysis of clinical finding, can help address the correct differential diagnosis (Table. 2).

We acknowledge that our study has limitations. Firstly, it is retrospective and enrolled a small number of patients. Secondly, the cohort studied was followed at our nephrology clinic, consequently there is a greater representation of patients with CKD; conversely, in the literature MSK is considered in most cases a benign disease that can lead to renal impairment only because of complicated and recurrent urinary tract infections or recurrent nephrolithiasis. Moreover, ten patients came from two families, in which the most severe cases (ESRD patients) were over-represented. So, the presence of CKD in a great percentage of our patients could be due both to familiar clustering of severe cases and to patients recruitment from a nephrology clinic. Finally, we lacked the IVU studies to confirm the diagnosis reported on medical records. Nevertheless, the review of CT images, available for 7 patients, by our expert radiologist, detected typical features of MSK in one patient according to what was reported in the studies by Koraishy [15] and Gaunay [16] and confirm the presence of cysts and calcifications in the other cases strengthening our diagnosis.

So, we believe that a thorough differential diagnosis and a critical appraisal of both clinical findings, history, biochemical analysis and imaging studies, can help make the diagnosis of MSK with good accuracy even in the absence of the gold standard imaging technique.

Even in the presence of the above biases, we think that the our preliminary study has important points of clinical relevance, namely: 1) given the diffuse availability of bedside US imaging, a wider use of our proposed ultrasonographic tetrad could increase the frequency of the recognition of MSK among patients with compatible clinical findings and avoid patient exposure to radiations and radiocontrast media; 2) US may offer a better follow-up for patients and relatives that need specific treatment of urinary tract infections and nephrolithiasis [1, 2, 5, 24]; 3) the possibility to intercept more diagnoses of MSK will allow to continue the research about its pathogenesis and specific treatment [25]; 4) it could encourage to explore the possibilities of ultrasound to define more and more specific characteristic of the disease.

## Conclusion

MSK is a rare kidney disease that is at risk to be underdiagnosed in the next future because urography is less and less used in the diagnostic work-up [12, 28]. After reviewing the results of US findings in our cohort, we believe that the diagnostic performance of US is greater than generally stated in the literature and that, when complemented with clinical findings, history and biochemical analysis, it may help establish the diagnosis of MSK [1, 2, 24]. Thus, promoting greater expertise of sonographers and imaging specialists in addressing specific MSK features could increase the number of correct diagnoses and provide patients with adequate follow-up and appropriate treatment.

## Supplementary information

**Additional file 1:Table S1.** Clinical data of the patients involved in the study.

**Additional file 2 :Table S2.** Clinical and instrumental features suggestive for nephrocalcinosis or ADTKD/MCKD.

## Data Availability

data were collected at our Centre and not deposited in a data repository, they are available as supplementary material.

## Abbreviations

MSK: Medullary sponge kidney
Tm: Transport maximum
IVU: Intravenous urography
CT: Computed tomography
MR: Magnetic resonance
eGFR: Estimated glomerular filtration rate
SD: Standard deviation
CKD: Chronic kidney disease
ESRD: End-stage renal disease
UTI: Urinary tract infection
US: Ultrasound
CTU: Computed tomographic urography
ADPKD: Autosomal dominant polycystic kidney disease
MCKD: Medullary cystic kidney disease
ADTKD: Autosomal dominant tobulointerstitial kidney disease

## References

[1] Gambaro G, Danza FM, Fabris A (2013). Medullary sponge kidney. Curr Opin Nephrol Hypertens.

[2] Fabris A, Anglani F, Lupo A, Gambaro G (2013). Medullary sponge kidney: state of the art. Nephrol Dial Transplant.

[3] Forster JA, Taylor J, Browning AJ, Biyani CS (2007). A review of the natural progression of medullary sponge kidney and a novel grading system based on intravenous urography findings. Urol Int.

[4] Gambaro G, Feltrin GP, Lupo A, Bonfante L, D'Angelo A, Antonello A (2006). Medullary sponge kidney (Lenarduzzi-Cacchi-Ricci disease): a Padua medical school discovery in the 1930s. Kidney Int.

[5] Castagna TL, Kim M (2005). Medullary sponge kidney: an imaging study. J Diagn Med Sonography.

[6] Imam TH, Patail H (2019). Medullary sponge kidney: current perspectives. Int J Nephrol Renov Dis.

[7] Garfield K, Leslie SW. Medullary Sponge Kidney. In: StatPearls [Internet]. Treasure Island (FL): StatPearls Publishing; 2020. 29262095

[8] Di Egidio G, Masciovecchio S, Saldutto P, Paradiso Galatioto G, Vicentini C (2014). Imaging of medullary sponge kidney: notes for urologists. Urologia..

[9] Palubinskas AJ (1961). Medullary sponge kidney. Radiology..

[10] Xiang H, Han J, Ridley WE, Ridley LJ (2018). Medullary sponge kidney. J Med Imaging Radiat Oncol.

[11] Teichman JM (2004). Clinical practice. Acute renal colic from ureteral calculus. N Engl J Med.

[12] Stratta P, Fenoglio R, Quaglia M, Lazzarich E, Airoldi A (2009). The missing medullary sponge kidney. Kidney Int.

[13] Ginalski JM, Schnyder P, Portmann L, Jaeger P (1991). Medullary sponge kidney on axial computed tomography: comparison with excretory urography. Eur J Radiol.

[14] Maw AM, Megibow AJ, Grasso M, Goldfarb DS (2007). Diagnosis of medullary sponge kidney by computed tomographic urography. Am J Kidney Dis.

[15] Koraishy FM, Ngo TT, Israel GM, Dahl NK (2014). CT urography for the diagnosis of medullary sponge kidney. Am J Nephrol.

[16] Gaunay GS, Berkenblit RG, Tabib CH, Blitstein JR, Patel M, Hoenig DM (2018). Efficacy of multi-detector computed tomography for the diagnosis of medullary sponge kidney. Curr Urol.

[17] Stephen J (2013). Medullary sponge kidney and medullary Nephrocalcinosis. J Diagnostic Med Sonography.

[18] Ferro F, Vezzali N, Comploj E, Pedron E, Di Serafino M, Esposito F (2019). Pediatric cystic diseases of the kidney. J Ultrasound.

[19] Dillman JR, Trout AT, Smith EA, Towbin AJ (2017). Hereditary renal cystic disorders: imaging of the kidneys and beyond. Radiographics..

[20] Wood CG, Stromberg LJ, Harmath CB, Horowitz JM, Feng C, Hammond NA (2015). CT and MR imaging for evaluation of cystic renal lesions and diseases. Radiographics..

[21] Katabathina VS, Kota G, Dasyam AK, Shanbhogue AK, Prasad SR (2010). Adult renal cystic disease: a genetic, biological, and developmental primer. Radiographics..

[22] Thomsen HS, Levine E, Meilstrup JW, Van Slyke MA, Edgar KA, Barth JC (1997). Renal cystic diseases. Eur Radiol.

[23] Stuck KJ, Koff SA, Silver TM (1982). Ultrasonic features of multicystic dysplastic kidney: expanded diagnostic criteria. Radiology..

[24] Fabris A, Lupo A, Ferraro PM, Anglani F, Pei Y, Danza FM (2013). Familial clustering of medullary sponge kidney is autosomal dominant with reduced penetrance and variable expressivity. Kidney Int.

[25] Torregrossa R, Anglani F, Fabris A, Gozzini A, Tanini A, Del Prete D (2010). Identification of GDNF gene sequence variations in patients with medullary sponge kidney disease. Clin J Am Soc Nephrol.

[26] Eckardt KU, Alper SL, Antignac C, Bleyer AJ, Chauveau D, Dahan K (2015). Autosomal dominant tubulointerstitial kidney disease: diagnosis, classification, and management--a KDIGO consensus report. Kidney Int.

[27] Stokman M, Lilien M, Knoers N (2016). Nephronophthisis.

[28] Goldfarb DS (2013). Evidence for inheritance of medullary sponge kidney. Kidney Int.

