# Supplementary material for: Ultrasound to address medullary sponge kidney: a retrospective study
Source: BMC Nephrol. 2020 Oct 12;21:430. doi: 10.1186/s12882-020-02084-1 (PMC7552549; doi:10.1186/s12882-020-02084-1)
Supplement: Supplementary file 1 — Additional file 1:Table S1. Clinical data of the patients involved in the study. [file 12882_2020_2084_MOESM1_ESM.docx]

**Supplementary table 1: Clinical data of the patients involved in the study**

| **Patient** | **Fam. History** | **Symptoms** | | | | **CKD** | **eGFR** | **HD** | **Tx** | **Comorbidities** |
| --- | --- | --- | --- | --- | --- | --- | --- | --- | --- | --- |
|  |  | **Proteinuria (Y/N)** | **Michrohematuria (Y/N)** | **UTI (Y/N)** | **Renal stones (Y/N)** |  |  |  |  |  |
| 1 | Y | Y | Y | N | N | Y | 34 | Y | Y |  |
| 2 | Y | Y | Y | Y | N | Y | 26 | Y | Y |  |
| 3 | Y | N | Y | N | N | N | 105 | N | N |  |
| 4 | Y | N | Y | N | Y | N | 117 | N | N |  |
| 5 | Y | Y | N | Y | N | Y | 20 | N | N |  |
| 6 | Y | Y | Y | N | N | Y | 18 | Y | N | DM; HTN |
| 7 | Y | Y | Y | N | N | Y | 49 | N | N |  |
| 8 | Y | Y | N | N | N | Y | 39 | N | N |  |
| 9 | Y | N | N | N | N | N | 116 | N | N |  |
| 10 | N | N | N | Y | Y | Y | 19 | N | N |  |
| 11 | U | N | N | N | Y | N | 111 | N | N |  |
| 12 | U | N | N | N | N | Y | 63 | N | N | Right nephrectomy |
| 13 | N | N | N | Y | Y | Y | 58 | N | N | DM |
| 14 | N | N | N | Y | N | Y | 85 | N | N |  |
| 15 | N | Y | Y | Y | Y | Y | 109 | Y | Y | DM |
| 16 | Y | NA | NA | NA | NA | Y | 29 | Y | N |  |
| 17 | N | N | Y | Y | N | N | 116 | N | N |  |
| 18 | N | Y | Y | Y | Y | Y | 38 | N | N |  |

Y: yes; N: no; NA: not available; Age: years at first evaluation at our Outpatient Clinic; UTI: urinary tract infection; DM: diabetes mellitus; HTN: hypertension; CKD: chronic kidney disease (either present at first evaluation at our Outpatient Clinic or developed during follow-up); eGFR: estimated glomerular filtration rate using CKD-EPI formula, ml/min/1.73m2 at first evaluation at our Outpatient Clinic; Tx: renal transplant; HD: hemodialysis.
