# Supplementary material for: Ultrasound to address medullary sponge kidney: a retrospective study
Source: BMC Nephrol. 2020 Oct 12;21:430. doi: 10.1186/s12882-020-02084-1 (PMC7552549; doi:10.1186/s12882-020-02084-1)
Supplement: Supplementary file 2 — Additional file 2 :Table S2. Clinical and instrumental features suggestive for nephrocalcinosis or ADTKD/MCKD. [file 12882_2020_2084_MOESM2_ESM.docx]

**Supplementary table 2: Clinical and instrumental features suggestive for nephrocalcinosis or ADTKD/MCKD**

| **Patient** | **Caracteristics of renal ultrasound** | | | | | **NPHP** | **ADTKD** | | | **Nephrocalcinosis** | | |
| --- | --- | --- | --- | --- | --- | --- | --- | --- | --- | --- | --- | --- |
|  |  |  |  |  |  |  | **UMOD** | **REN** | **HNF1β** |  | | |
|  | **Renal size**  **(cm)** | **Cyst localization**  **(unilateral/**  **bilateral)** | **Cyst size (diameter)** | **Cyst distribution** | **Renal stones**  **/calcifications**  **(Yes/No)** | **Polyuria / polydipsia**  **(Yes/No)** | **Hyperuricemia / gout**  **(Yes/No)** | **Anemia**  **(Yes/No)** | **Mody5**  **(Yes/ No)** | **Hypercalcemia**  **(Yes/No)** | **Elevated serum PTH**  **(Yes/No)** | **Vitamin D supplement**  **(Yes/**  **No)** |
| 1 | NA | bilateral | A cortical cyst of 65 mm  The others are mychrocysts | parenchimal | Yes | No | Yes | Yes | No | No | Yes | No |
| 2 | R 9,5; L9,5 | bilateral | Cortical: 8-33 mm  Medullary: 5-30 mm | cortical and medullary | Yes | No | Yes | Yes | No | No | Yes | No |
| 3 | R 11; L 11,1 | bilateral | Up to 11 mm | parenchimal | Yes | No | No | No | No | No | No | No |
| 4 | R 14; L14,9 | bilateral | Up to 30 mm | medullary | Yes | No | No | No | No | No | No | No |
| 5 | R 9,7; L 9,6 | bilateral | 4-10 mm | medullary | Yes | No | No | Yes | No | No | Yes | No |
| 6 | R 10; L10 | bilateral | Up to 40 mm | parenchimal | Yes | No | No | No | No | No | Yes | No |
| 7 | R 8,5; L 7,8 | bilateral | 1-8 mm  Cortical cyst: 20x27 mm | medullary with a cortical cyst | Yes | No | Yes | Yes | No | No | No | No |
| 8 | Normal | bilateral | Up to 19 mm | cortico-medullary | No | No | No | Yes | No | No | Yes | No |
| 9 | R 11,3; L 10,9 | bilateral | 3-4 mm | medullary | Yes | No | No | Yes | No | No | No | No |
| 10 | R 8,7; L 9,7 | bilateral | 2-12 mm | medullary | Yes | No | No | No | No | NA | NA | NA |
| 11 | R 11,5; L 11,9 | bilateral | NA | medullary | Yes | No | No | No | No | NA | NA | NA |
| 12 | L 14 | unilateral | Cortical: up to 17 mm  Medullay: up to 13 mm | cortical and medullary | Yes | No | No | No | No | No | NA | No |
| 13 | Normal | bilateral | Up to 22 mm | NA | No | No | No | No | No | No | No | No |
| 14 | R 11,1; L 12,1 | bilateral | 3-5 mm | medullary | Yes (Nephrocalcinosis) | No | No | No | No | No | No | No |
| 15 | R 10,8; L 11,1 | bilateral | Michrocysts | medullary | Yes | No | No | No | No | No | No | No |
| 16 | Small | bilateral | NA | cortical | Yes | No | No | No | No | No | No | No |
| 17 | NA | unilateral | Up to 15 mm | NA | Yes | No | No | No | No | No | No | No |
| 18 | R 8,9; L 9.5 | NA | NA | NA | Yes | No | No | No | No | No | No | No |

NA: not available; R: right kidney; L: left kidney; NPHP: nephronoptisis; ADTKD: autosomal dominant tubulointerstitial kidney disease.
